# Evaluation of multiple approaches to identify genome-wide polymorphisms in closely related genotypes of sweet cherry (*Prunus avium* L.)

**DOI:** 10.1016/j.csbj.2017.03.002

**Published:** 2017-03-18

**Authors:** Seanna Hewitt, Benjamin Kilian, Ramyya Hari, Tyson Koepke, Richard Sharpe, Amit Dhingra

**Affiliations:** a Molecular Plant Sciences Graduate Program, Washington State University, Pullman, WA 99164, United States; b Department of Horticulture, Washington State University, Pullman, WA 99164-6414, United States

**Keywords:** Polymorphisms, *Prunus avium*, Next-generation sequencing, Target region amplification polymorphism (TRAP), Genetic diversity, SNParray, Reduced representation sequencing, Whole genome sequencing (WGS)

## Abstract

Identification of genetic polymorphisms and subsequent development of molecular markers is important for marker assisted breeding of superior cultivars of economically important species. Sweet cherry (*Prunus avium* L.) is an economically important non-climacteric tree fruit crop in the Rosaceae family and has undergone a genetic bottleneck due to breeding, resulting in limited genetic diversity in the germplasm that is utilized for breeding new cultivars. Therefore, it is critical to recognize the best platforms for identifying genome-wide polymorphisms that can help identify, and consequently preserve, the diversity in a genetically constrained species. For the identification of polymorphisms in five closely related genotypes of sweet cherry, a gel-based approach (TRAP), reduced representation sequencing (TRAPseq), a 6k cherry SNParray, and whole genome sequencing (WGS) approaches were evaluated in the identification of genome-wide polymorphisms in sweet cherry cultivars. All platforms facilitated detection of polymorphisms among the genotypes with variable efficiency. In assessing multiple SNP detection platforms, this study has demonstrated that a combination of appropriate approaches is necessary for efficient polymorphism identification, especially between closely related cultivars of a species. The information generated in this study provides a valuable resource for future genetic and genomic studies in sweet cherry, and the insights gained from the evaluation of multiple approaches can be utilized for other closely related species with limited genetic diversity in the breeding germplasm.

## 1 Introduction

Plants are fundamental to continued life on this planet as they are the basis of food production and an essential part of the global ecosystem. Application of different molecular tools and access to plant genomes has facilitated identification of genome-wide polymorphisms and thus, development of molecular markers that can be utilized in breeding programs [1], [2]. Next-generation sequencing now allows genomic information to be obtained, even for non-model plant systems, further accelerating the development of molecular markers and genetic research [3], [4]. Efforts to efficiently develop desirable genotypes by establishing an association of important agronomic traits, such as yield, nutritional content, and timing of flowering and fruit ripening with specific polymorphic regions of the genome, are ongoing in various plant species [5], [6].

Sweet cherry (*Prunus avium* L.) is a member of the Rosaceae family, which represents many other important crop species, including apple, peach, plum, almond, strawberry, raspberry and rose [7]. Despite an estimated genome size of 225–330 Mb [8], [9], sweet cherry is lacking in genomic information in comparison with other prominent Rosaceae members, including peach and apple [10], [11]. Linkage maps and molecular markers have been developed for sweet cherry [12] as well as peach and almond, two other members of the sub-family Prunoideae [13], [14], [15], and a comprehensive and advanced draft of the peach genome serves as the foundation for several comparative studies [10]. Recently, a draft genome of sweet cherry cultivar ‘Stella’ was released [16]. To advance diversity and genetics-related studies, efforts were made to evaluate the transferability of the molecular markers from one member of Rosaceae family to other members with mixed success [17], [18], [19].

In addition to lack of comprehensive genetic information, domesticated sweet cherry cultivars exhibit a genetic bottleneck as a result of breeding. Despite the prevalence of several wild landraces [20], there are only three chloroplast haplotypes represented in the commercial cultivars indicating a very narrow maternal parental lineage in sweet cherry [21], [22]. Given the genetic closeness, it can be difficult to identify genetic diversity unless comprehensive approaches are utilized. A recent study in tree genus *Milica*, where population structure was analyzed using nuclear SNPs, SSRs and DNA sequences, revealed hidden species diversity in closely related species [23]. In sweet cherry, a previous study compared and evaluated the utility of 7 simple sequence repeat (SSR) molecular markers versus 40 single nucleotide polymorphism (SNP) molecular markers to determine the genetic diversity and relatedness in 99 cultivated genotypes of sweet cherry [24]. SSRs were found to generate a higher average number of alleles per locus, mean observed heterozygosity, expected heterozygosity, and polymorphic information content values; however, the SNPs allowed for finer resolution of a closely related genotype, which was indistinguishable with SSRs. Despite the higher resolution of SNPs, both sets of markers produced a similar genetic relatedness for all the accessions tested [24].

In this study, the efficiency of different genotyping approaches was evaluated to differentiate between five sweet cherry cultivars. The cultivars selected for diversity analysis are suspected to be very closely related, and their interrelatedness was not tested in the previous study that included 99 cultivars [24]. The genotypes included a newly identified cultivar named ‘Glory,’ which was proposed to be an open-pollinated seedling of ‘Sonata’. However, it has also been proposed that it is the same cultivar as ‘13S2009’ ‘Staccato’, owned by Summerland Variety Corporation, Canada [25], [26], [27]. Similarly, ‘Kimberly’ and ‘Bing’ were selected since it has been proposed that the former may have been derived from the latter as a random mutation or sport [28]. ‘Sweetheart’ was selected as it is the parent of ‘Staccato’ [29]. The newly released cultivars ‘Glory’ and ‘Kimberly’ represent late maturing cultivars, like ‘Staccato’ and ‘Sweetheart’, making them highly desirable cultivars. The similarity in late maturing phenotype across the four cultivars has led to the notion that the new cultivars may share a close genetic relationship, or that they may in fact be the same as previously released cultivars. In order to resolve the identity conundrum and understand the genetic relationship between these cultivars and genetically distinguish them from each other, a gel-based, Targeted Region Amplified Polymorphism (TRAP) approach [30], a reduced representation or genotype by sequencing (GBS) approach called TRAPseq, a *Prunus* SNParray [31], and a whole genome sequencing (WGS) approach were evaluated for their relative effectiveness.

## 2 Methods

### 2.1 Plant Material Source and Preparation

Five sweet cherry genotypes used in this study were obtained from VanWell Nursery, East Wenatchee, WA. Emerging leaf samples were collected for each genotype following fruit harvest and flash frozen in liquid nitrogen. All samples were pulverized under liquid nitrogen using SPEX SamplePrep® FreezerMill 6870 (Metuchen, NJ, USA) and kept frozen at − 80 °C prior to processing.

### 2.2 Genomic DNA Extraction

Total genomic DNA was extracted from young leaf tissue using cetyltrimethylammonium bromide (CTAB) phenol chloroform extraction method [32]. Extracted DNA pellets were air dried and suspended in 50 μl of nuclease-free water and incubated at 37 °C with DNase free RNAse for 30 min. RNAse was inactivated by incubating the tubes at 65 °C for 10 min. DNA was quantified using Nanodrop 8000 spectrophotometer (Thermo Scientific, Waltham, MA, USA) and 50 ng of extracted genomic DNA was electrophoresed on a 1% agarose gel and compared to Lambda DNA dilution series (100, 80, 60, 40, 20, 10 ng) to confirm quality and quantity.

### 2.3 TRAP — Target Region Amplification Polymorphism

PCR was conducted with a final reaction volume of 10 μl in a BioRad ICycler (Bio-Rad Laboratories, Hercules, CA) with components in the following final concentrations: 10 ng DNA, 1.5 mM MgCl_2_, 0.2 mM dNTPs, 0.02 mM 700- and 800-IR dye-labeled arbitrary primers, 0.2 mM fixed primer (BRK 393 or BRK 394, Table 1), and 1 U Taq DNA polymerase and 1 × corresponding polymerase buffer (Biolase). PCR was carried out by initially denaturing the template DNA at 94 °C for 2 min. The thermocycle profile consisted of five cycles of 94 °C for 45 s, 35 °C for 45 s, and 72 °C for 1 min, followed by 35 cycles at 94 °C for 45 s, 50 °C for 45 s, and 72 °C for 1 min. The final extension step was at 72 °C for 7 min. Thereafter, 5 μl of IR stop dye was added and the product was denatured at 4 °C for 4 min. A 6.5% polyacrylamide gel (KB-PLUS, LI-COR) was cast, the reactions loaded, and the PCR product electrophoresed at 1500 V for 2.5 h in a Li-COR 4300 DNA Analyzer (LI-COR Biosciences, Lincoln, NE). Images were captured by the Li-COR instrument and analyzed using LI-COR 4300 DNA Analyzer image software to identify polymorphisms.

### 2.4 TRAPseq and Read Processing Using Stacks and BLAST2GO Analysis

Genomic DNA (~ 1 μg) was isolated from ‘Glory’ and ‘Staccato’ young leaf tissue. The reduced representation of the genome was achieved by performing TRAP PCR with fixed primers targeting MADS-box, PPR1, and PPR2 gene families (Table 1). Amplification was followed by generation of NGS sequence data from the products (Ion Torrent PGM, Thermo Fisher Scientific, Inc., Waltham, MA). The short read sequence data generated from TRAPseq was submitted to NCBI under the following accession numbers: SRS1706064 - Glory_Trapseq and SRS1706056 - Staccato_Trapseq. The fixed MADS primer was selected because the MADS-box gene family is predicted to contain polymorphic regions even in closely-related plant cultivars [33], [34]. The TRAP PCR parameters used were identical to the TRAP protocol described above, except for the 5-min denaturing step. Following TRAP amplification and PCR cleanup, the reduced representation sample library was prepared using the NEBNext® Fast DNA Library Prep Set as per the manufacturer's instructions with the following modifications. TRAP PCR products from each reaction were sheared with NEB Next Fragmentase. After heat disabling the fragmentase, each sample was processed for A-tailing by adding 0.2 mM dATP (1 mM stock), 1 U of Taq polymerase (5 U/μl), 1.6 mM of MgCl_2_ (50 mM), and 1 × Taq polymerase buffer (10 × stock). Complementary, custom adaptors were then annealed to the sheared DNA, the annealed product was purified and extracted according the NEBNext FastDNA Library Prep protocol. The libraries were quantified, pooled, and sequenced using the Ion Torrent PGM (Life Technologies, Inc.). The sequencing run included 850 flows on a 318C chip producing single reads of various lengths.

The sequenced libraries (Ion Torrent PGM, Thermo Fisher Scientific, Inc., Waltham, MA) generated ~ 230 Mb combined data for ‘Glory’ and ‘Staccato’ genotypes, comprised of 795k reads with an average read length of 145 nucleotides. The sequencing data was processed through the Stacks program to identify loci containing polymorphisms [35]. This allowed for the generation of an output file containing the Stacks catalog ID and corresponding genotype for ‘Glory’ and ‘Staccato’ at each locus (Supplementary File 1). Each combination of nucleotides at the polymorphic loci was assigned a numeric code of 1–16. All loci originally identified in Stacks were run through the Blast2GO sequence alignment, gene ontology (GO) mapping, and functional annotation pipeline [36], [37]. The output file is available as Supplementary File 2. Sequences were processed through BLAST against the Viridiplantae database using an e-value cutoff of 1.0e − 3 [38].

### 2.5 SNParray

For this experiment, ‘Bing’, ‘Sweetheart’, ‘Glory’, ‘Kimberly’ and ‘Staccato’ sweet cherry cultivars were analyzed using the sweet cherry 6k Infinium II SNParray [31]. The output data were analyzed with GenomeStudio v. 1.0, Genotyping module (Illumina, Inc., San Diego, CA), which determines cluster positions of the AA/AB/BB genotypes for each putative SNP. Default quality metrics for GenomeStudio were used in the assay: GenTrain score ≥ 0.5, minor allelic frequency (MAF) ≥ 0.15 and call rate of > 80%. The resulting data show pair-wise comparisons between each cultivar for each specific SNP. A subset of the predicted SNPs was evaluated in silico by using BLAST to compare twenty SNPs from NCBI with the de novo assembly from each genotype. All twenty SNPs tested were confirmed using this method (Supplementary File 3).

The identified SNPs were filtered to remove missing data, assigned numeric codes corresponding to respective AA/AB/BB genotype, and categorized for downstream population structure analysis.

### 2.6 WGS and Genetic Diversity Analysis Using Stacks

For all the genotypes, approximately 25 × coverage sequence data represented by 2 × 100 paired end reads, were generated with the Illumina HiSeq 2000 sequencing platform. All short read sequenced data was submitted to NCBI under the following accession numbers: SRS1706059 - Bing_Illumina; SRS1706061 - Sweetheart_Illumina; SRS1706060 - Staccato_Illumina; SRS1706062 - Glory_Illumina; SRS1706063 - Kimbery_Illumina. Stacks [35] was used to identify SNPs from the short-read sequence genomic data. This was accomplished through building artificial loci from the raw data (‘stacks’ of reads). An internal module (Process_shortreads) was used which filters reads with uncalled bases, discards reads with low quality scores and removes any traces of remaining inline barcodes. Thereafter, the dataset was processed by running the de novo map wrapper, which includes ustacks, cstacks, sstacks, populations (map). Ustacks builds stacks, forms loci, and looks for SNPs. Cstacks merges identified loci together across a population based on the consensus sequence from each locus. Then, sstacks creates a map between the loci in the population that match the catalog and assigns respective catalog IDs to these loci [35]. SNPs were detected at each locus using a maximum likelihood framework by iteratively comparing loci for each sweet cherry genotype in a pairwise comparison against other genotypes.

### 2.7 Population Structure Analysis Using STRUCTURE and NTSys

A SNP-based population structure analysis was conducted for both the SNParray and the Stacks data using STRUCTURE [39] and NTSys [40]. Loci with missing data were omitted from the final analyses, as were loci with the same score for each of the 5 genotypes. For the SNParray data, the cherry genotypes were assigned a numeric code of 1–6, corresponding to the respective AA/AB/BB genotype at each polymorphic locus. This was the input file for the subsequent STRUCTURE analysis (Supplementary File 4). For the WGS data, a structure.tsv file from the Stacks ‘populations’ output was modified. Numbers 1, 2, 3, and 4 were used to code for A, C, G, and T, respectively, and ‘0’ was used to indicate missing data. The Stacks output file contained information regarding the replicates and separate paired end reads for each allele, therefore, to consolidate data, the most frequent non-zero nucleotide code was identified for each genotype (Supplementary File 5, Supplementary File 6). The modified SNParray and WGS Stacks files were saved as *.csv files for input into STRUCTURE (Supplementary File 7). The parameters for the preparation of data upload to STRUCTURE were as follows: row of marker names = TRUE, individuals = 5, ploidy = 2, loci = 9029. Additional parameters for running the population structure algorithm were specified as follows: Length of Burnin Period = 20,000, Number of MCMC Reps after Burnin = 20,000, Use Admixture Model = TRUE, Allele Frequencies Correlated = TRUE, Compute probability of the data (for estimating K) = TRUE, Print Q-hat = TRUE.

Analysis of K values from 1 to 5 was specified, along with 5 iterations of the defined STRUCTURE analysis. Upon completion of the Structure run, Structure Harvester was used for identification of most likely K-value based on the data [41].

The NTSys software [40] was used to produce a tree dendrogram and to determine sample order for the population structure output. The latter is used for running of CLUMPP [42] and DISTRUCT [43] clustering and visualization programs. The SNParray and WGS data files for input into NTSys were prepared by modifying the STRUCTURE files (Supplementary File 8, Supplementary File 9). In the case of the Stacks data, the alleles were assigned an ID of ‘a’ or ‘b’ and were listed under their respective genotypes to be treated as separate markers in the NTSys analysis. This was not necessary for the SNParray data, as the allele combinations were assigned numeric codes, as previously stated.

To run NTSys, the input files were uploaded, and the following functions run: 1.) Qualitative data Dis/Similarity method, 2.) SAHN UPGMA clustering method 3.) Tree plot graphic generation function. The result is a tree dendrogram representing WGS SNP-based genetic relationships (Fig. 6). The K2 and K4 indfiles from the Structure Harvester output were then run through CLUMPP and DISTRUCT [42], [43] according to an in-house workflow to produce a graphic representing population structure.

### 2.8 Validation of NTSys and STRUCTURE Results

To validate the NTSys and STRUCTURE outputs, Excel was used to calculate the number of SNPs in pairwise comparisons between each genotype, with Bing as the reference genotype. The resulting data was prepared as a distance matrix— genetic distance (or genotypic variation) increases as the number of SNPs increases.

The data was saved as a *.txt file and imported into R studio as a “dist” object for further analysis. A dendrogram similar to the one generated by NTSys was produced using the R “plot” and “hclust” functions. As with NTSys, the UPGMA (“average”) method of hierarchical clustering was employed to generate a Euclidian distance-based tree dendrogram which could be compared to the results of the NTSys output (Supplementary File 10, Supplementary File 11).

## 3 Results and Discussion

### 3.1 Pedigree Information of the Five Genotypes and Genomics Approaches Evaluated

**Fig. 1:**
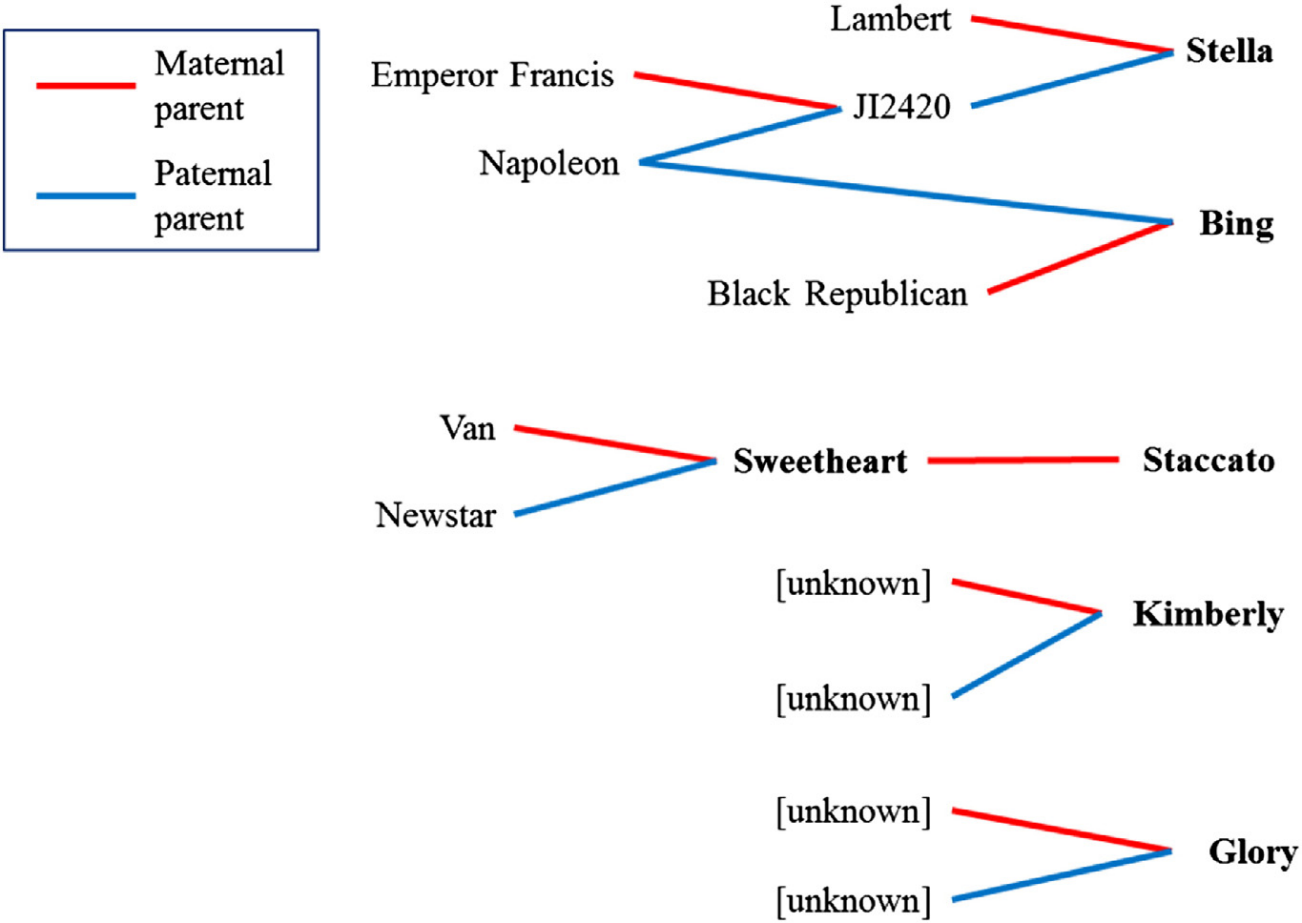
Pedigree relationships of five of the sweet cherry cultivars analyzed in this study Pedigree of the sweet cherry cultivars used for SNP development. The maternal parent is marked by a red line and the parental parent by a blue line.

Given the documented lack of genetic diversity within the cultivars of sweet cherry, it is important to understand the pedigree information regarding the five genotypes used in this study namely, ‘Bing’, ‘Sweetheart’, ‘Staccato’, ‘Glory’ and ‘Kimberly’. ‘Sweetheart’ is known to be the maternal parent of ‘Staccato’ while the paternal parent is unknown as it was developed via open pollination. ‘Van’ and ‘Newstar’ (pollinator) are the parents of ‘Sweetheart’, but ‘Sweetheart’ and ‘Staccato’ have no known familial relationship to the other three genotypes used in this study. Previously published SNP marker analysis has shown the paternal parent of ‘Bing’ to likely be ‘Napoleon’ [44]. ‘Napoleon’ is also the paternal grandparent of ‘Stella’ (Fig. 1). Therefore, ‘Bing’ and ‘Stella’, for which the reference genome is available, share Napoleon in their pedigree as a paternal parent and grandparents respectively. ‘Kimberly’ and ‘Glory’ were serendipitous discoveries in orchards based on their delayed fruit maturation phenotype and therefore have unknown lineage. Three of the known sweet cherry cultivars used for analysis in this study belong to different self-incompatibility S-allele genotypes [45].

The first approach, TRAP assay, is a PCR-based technique that uses one fixed primer targeting a conserved DNA sequence usually representing a gene family across the genome and one or two arbitrary primers with either an AT- or GC-rich core that anneal to an intron or an exon, respectively [30]. The 5′ end of the arbitrary primers is fluorescently labeled to enable laser-mediated detection of DNA fragments during electrophoresis and subsequent polymorphism identification. Since it has been proposed that ‘Glory’ and ‘Staccato’ are the same genotypes, this approach was first employed to evaluate if there are any differences between the two genotypes using fixed primers targeting the flowering-related genes as based on shared ontogeny with the process of fruit development such genes may influence time of fruit maturation. The second approach, TRAPseq was developed as part of this study and is a modified reduced representation sequencing method derived from the TRAP assay. This method was also tested for its capacity to identify any differences between ‘Glory’ and ‘Staccato’. In the third approach, all five genotypes were analyzed using a sweet cherry SNParray. This 6K Infinium II array contains 5696 predicted genome-wide SNPs, 4214 from diploid sweet cherry (*P. avium*) and 1482 from allotetraploid sour cherry (*P. cerasus*) accessions [31]. For the final, and the highest-resolution approach, WGS was performed on the five genotypes followed by processing of short reads and identification of polymorphisms using Stacks [35]. Subsequent population structure analyses were performed using the SNParray data and Stacks output from the WGS data to determine the genetic relatedness of the genotypes based on the identified SNPs.

### 3.2 Evaluation of Gel-based Approach, TRAP

**Fig. 2:**
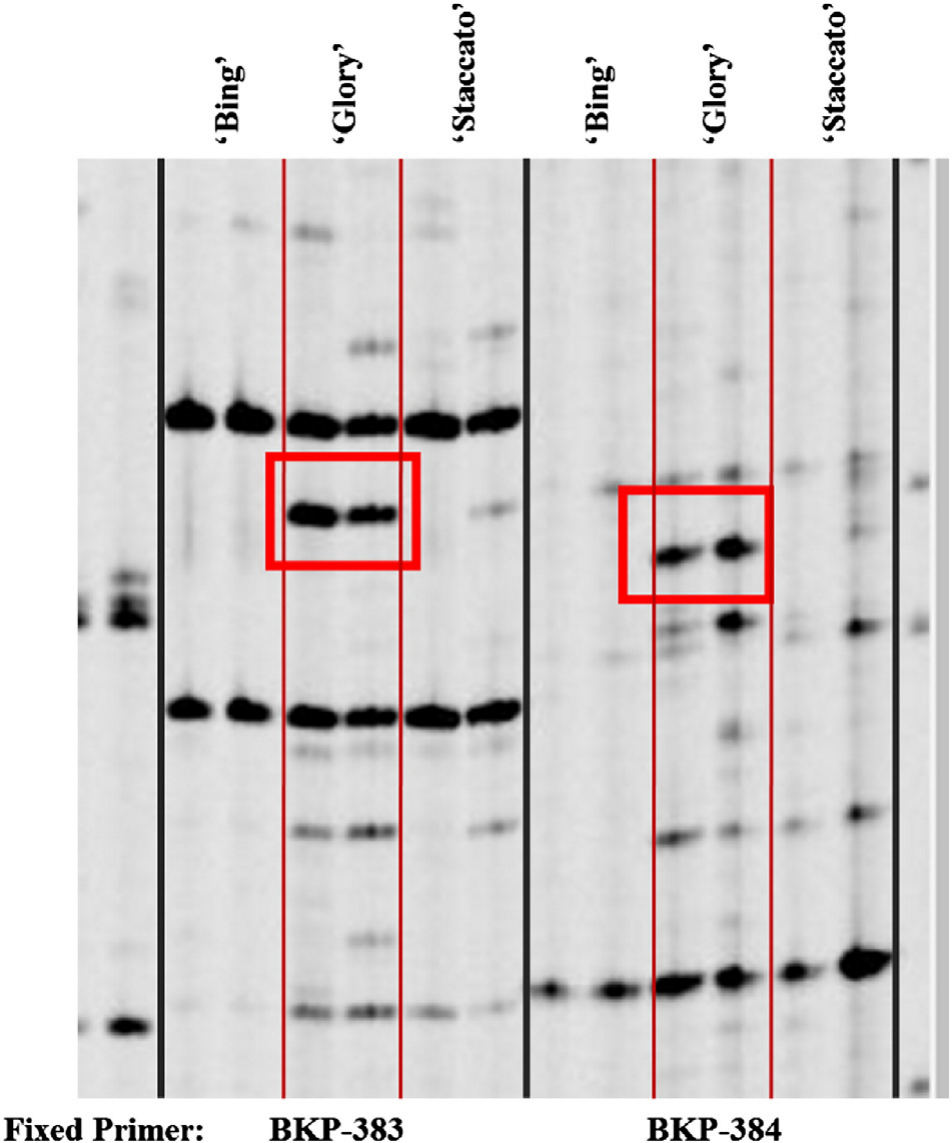
TRAP analysis of Bing, Glory and Staccato sweet cherry cultivars. Experiment was performed in duplicate. Primer screen was performed using fixed primers BKP-383, 384 and arbitrary primers SA12, GA5. Primer sequences are provided in Table 1. Red boxes are indicative of polymorphic loci. The size of the unique BKP-383 and BKP-384 ‘Glory’ amplicons is approximately 336 and 330 bp, respectively.

By specifically targeting a flowering-related gene family, we were able to identify polymorphisms between ‘Glory’ and ‘Staccato’ using the TRAP approach [30]. The fixed primer targeted the VRN2 gene, which has been implicated in temperature-induced induction of flowering [46], [47]. Two polymorphic regions were identified out of a total of 45 amplified loci (Table 1, Fig. 2). This corresponds to a 4.4% rate of polymorphism detection (Table 3). It is important to consider, however, that selection of fixed primer targets is particularly important when analyzing highly similar genotypes. As delayed maturation of the fruit is the only observable phenotypic difference between ‘Glory’ and ‘Staccato,’ TRAP primers were designed to target flowering related genes with the presumption that during the ontogenic progression, these genes may influence fruit maturation. Relationship between VRN2 and Polycomb-group Proteins, which work in concert to regulate fruit maturation in tomato has been reported recently [48]. It is premature to comment on the direct role of VRN2 in regulating fruit maturation in non-climacteric sweet cherry based on this result. However, when non-flowering gene-targeted primers were used no polymorphisms were detected (data not shown). This speaks to the utility of TRAP as a cost-effective and preliminary method for identification of genome wide polymorphisms only when fixed primers are specifically targeted to putative genes underlying an observable phenotype. While this method is the easiest to implement, it is a low-throughput approach that requires prior information about the trait and putative genes that may underlie the observable phenotype. TRAP is an empirical approach that may have limited success in identifying polymorphic loci since it each primer set provides access to a very small fraction of the genome.

### 3.3 Evaluation of TRAPseq — Modified Reduced Representation Sequencing to Identify Polymorphisms

The reduced representation of the genome was achieved by performing TRAP PCR, followed by generating NGS sequence data from the amplified products. By applying the Stacks pipeline and populations map to the TRAPseq data, 942 polymorphic loci corresponding to SNPs between ‘Glory’ and ‘Staccato’ out of 24,984 total loci were identified (Supplementary File 1). This corresponds to a 3.8% rate of polymorphism detection, slightly less than the polymorphism detection rate of the gel-based TRAP analysis, but more representative of genome-wide polymorphisms (Table 3). In terms of genome representation, TRAPseq accessed 0.01% of the genome whereas TRAP only accessed 0.0002% of the genome and that too without any sequence information. These results indicate the importance of identifying appropriate target genes for the fixed primer. While somewhat of a high-throughput approach, it provides a limited coverage of the genome. To enhance coverage, multiple primer sets may need to be utilized. One could utilize the TRAP gel approach to first assess the primer sets that provide the most polymorphic loci and then utilize the same primer sets for TRAPseq to enhance the identification of the number of polymorphic loci.

The Blast2GO gene annotation suite was used to identify the top NCBI Blast hit corresponding to each of the polymorphic loci identified via the TRAPseq analysis. Among the annotated loci were: G-type lectin S-receptor-like serine threonine- kinases, which have been implicated in drought, salinity and cold tolerance [49], ATPase WRNIP1(ATXAB2), which may play a role in DNA UV damage repair [50], [51], HIPP proteins, which are responsive to cold and drought conditions [52], SKP1 proteins, previously implicated in cell cycle progression and floral organ development [53], [54], DES1 protein homologues, which may interact with FLC in *Arabidopsis* to regulate flowering time [55], and succinate dehydrogenase complex subunit coding genes. As these sequences were identified via processing of short reads using Stacks, and were not extensive in length, increased stringency parameters ensured that only sequences of highest similarity to their top blast hit (e-value cutoff of 1.0e − 3) were annotated. In the case of ‘Glory’ v. ‘Staccato’, where delayed fruit maturity is the only observable difference at the phenotypic level, it is promising that several polymorphic sequences were identified in genes associated with flowering time, cold induction of developmental processes, and floral organ development. While further investigation is necessary to correlate the annotated gene fragments with the delayed fruit maturity phenotype between ‘Glory’ and ‘Staccato’, this analysis has demonstrated that functional annotation of polymorphic sequences can be of use in further understanding the genetic basis for phenotypic differences.

### 3.4 Evaluation of Cherry SNParray

SNParray analysis enabled the identification of 1385 polymorphic loci out of the 5696 representative loci in the five cultivars namely ‘Bing’, ‘Sweetheart’, ‘Glory’, ‘Kimberly’, and ‘Staccato’. This corresponds to a 24.3% SNP detection rate. The SNParray has been used previously to genotype sweet cherry cultivars and determine their genetic relatedness [24]. The putative polymorphisms represented on the array are spread relatively evenly across each chromosome, but their finite number derived from a pre-selected set of genotype indicates that only a representative subset of potential SNPs can be examined from the sweet cherry genome. Since the SNParray represents a limited number of SNPs derived from the originally represented genotypes, the efficacy of polymorphism detection is far greater for the represented genotype ‘Bing’. Approximately 600 SNPs were identified when ‘Glory’ and ‘Staccato’, were compared to ‘Bing’ however, only 66 SNPs were identified when the two genotypes were compared to ‘Sweetheart’ and ‘Kimberly’. The SNParray failed to detect any SNPs between ‘Glory’/‘Staccato’ and ‘Sweetheart’/‘Kimberly’ (Table 2). Furthermore, 174 unique SNPs (3.1%) were detected for ‘Bing’, whereas no unique SNPs were detected for ‘Glory’, ‘Staccato’, ‘Sweetheart’, or ‘Kimberly’ (Table 3). While a SNParray is a great analysis tool for repeat polymorphism detection in reference genotypes or samples that were originally represented on the array, it does have some major limitations when the target sample is different from the references sample set. The latter situation leads to the introduction of ascertainment bias [56] a statistical term that describes the deviation observed between real results versus expected results due to the use of non-reference samples. While there are approaches to overcome ascertainment bias, they may not be applicable in non-model plant systems as they lack vast amount of genomic data across the genera as in case of model systems.

### 3.5 Evaluation of WGS to Identify Polymorphisms

**Fig. 3:**
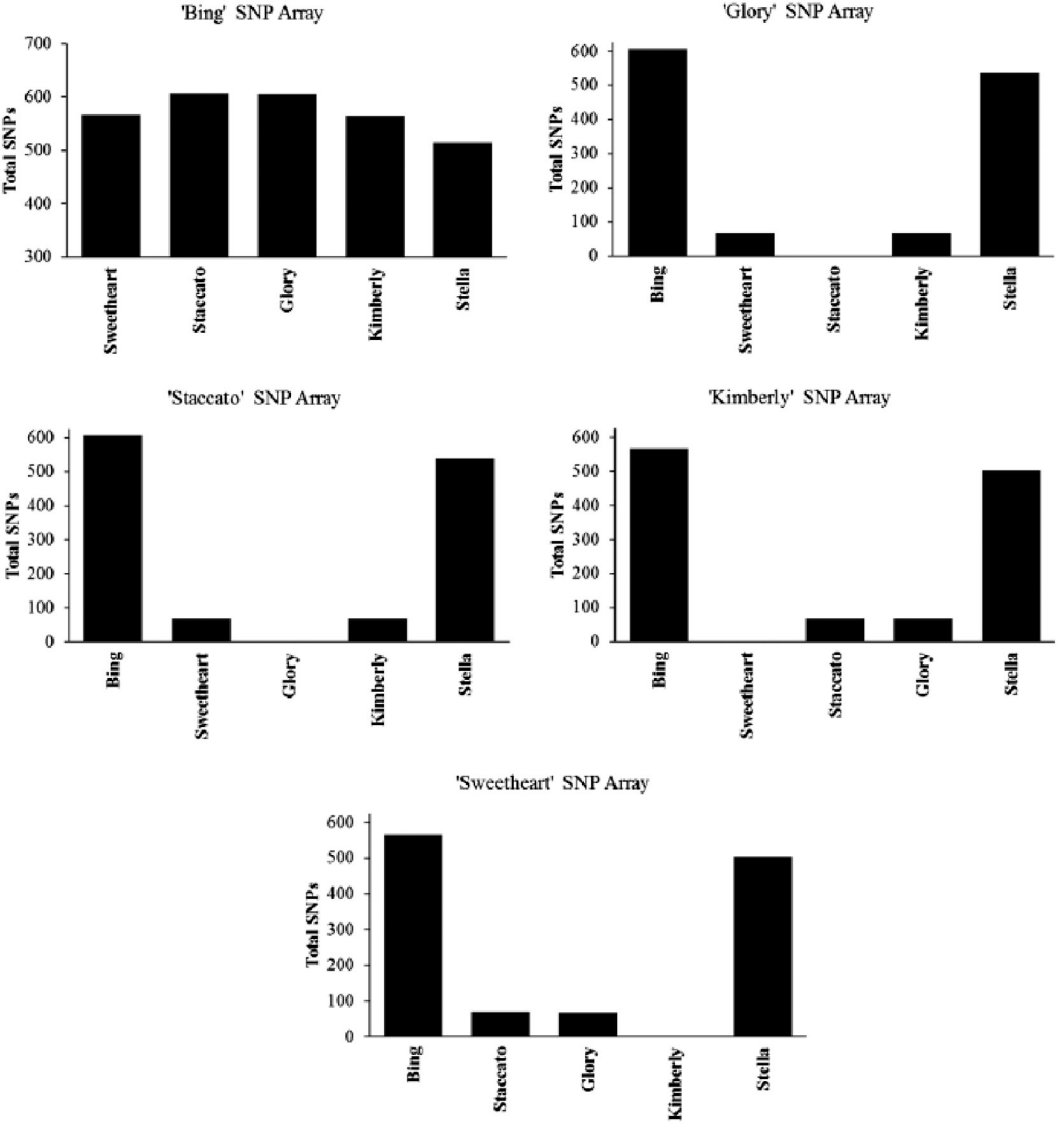
SNParray, individual genotype comparisons of total SNPs. The title of each subfigure indicates the reference by which the other genotypes were compared.

For each of the five genotypes analyzed using SNParray, 22.2 × average coverage of Illumina HiSeq paired end read data, or 4.6–5.5 Gb of sequence data were generated. SNPs were identified using the Stacks workflow [35], [57]. Stacks generated loci from short read Illumina data and identified polymorphisms within the genotype-specific loci. Overall, 2071 polymorphic loci were identified among the compared genotypes out of 1,239,693 catalog loci matching the generated stacks representing 0.5% of the sweet cherry genome. STRUCTURE analysis and subsequent identification of most probable ΔK values, representing population number, using STRUCTURE Harvester's Evanno method calculations revealed increased ΔK values at 2 and 4, indicating that there are four genetically distinct sweet cherry subgroups within two larger groups (Fig. 5). In both cases, ‘Bing’ segregated into its own group and subgroup. The final graphics files produced by DISTRUCT can be seen in Fig. 3, combined with the dendrogram produced by NTSys (Fig. 6).

While WGS enables the largest coverage of the genome, sequencing of random regions reduces the comparable areas across samples. Perhaps enhancing the depth of coverage can alleviate this limitation. The major strength of all sequencing based approaches over SNParray is that it directly couples SNP discovery with genotyping by identification of genome wide polymorphisms directly in the target samples.

The Blast2GO gene annotation suite was also used to identify the top NCBI Blast hit corresponding to each of the polymorphic loci identified by the WGS. The annotated loci included: RNA-directed DNA polymerases, receptor kinases, which have been implicated in brassinosteroid signaling [58], and numerous genes encoding plastid targeted proteins--NADH dehydrogenase subunits, NAD(P)H quinone oxidoreductase subunits, Rubisco subunits, and cytochrome b6 f complex precursors (Supplementary File 12). A large portion of the identified genes corresponding to polymorphic sequences are both plastid-targeted and plastid-encoded in nature. This is intriguing considering there are only three maternal haplotypes reported for all sweet cherry cultivars [21].

### 3.6 Comparison of Population Structures Derived from WGS and SNParray Data

**Fig. 4:**
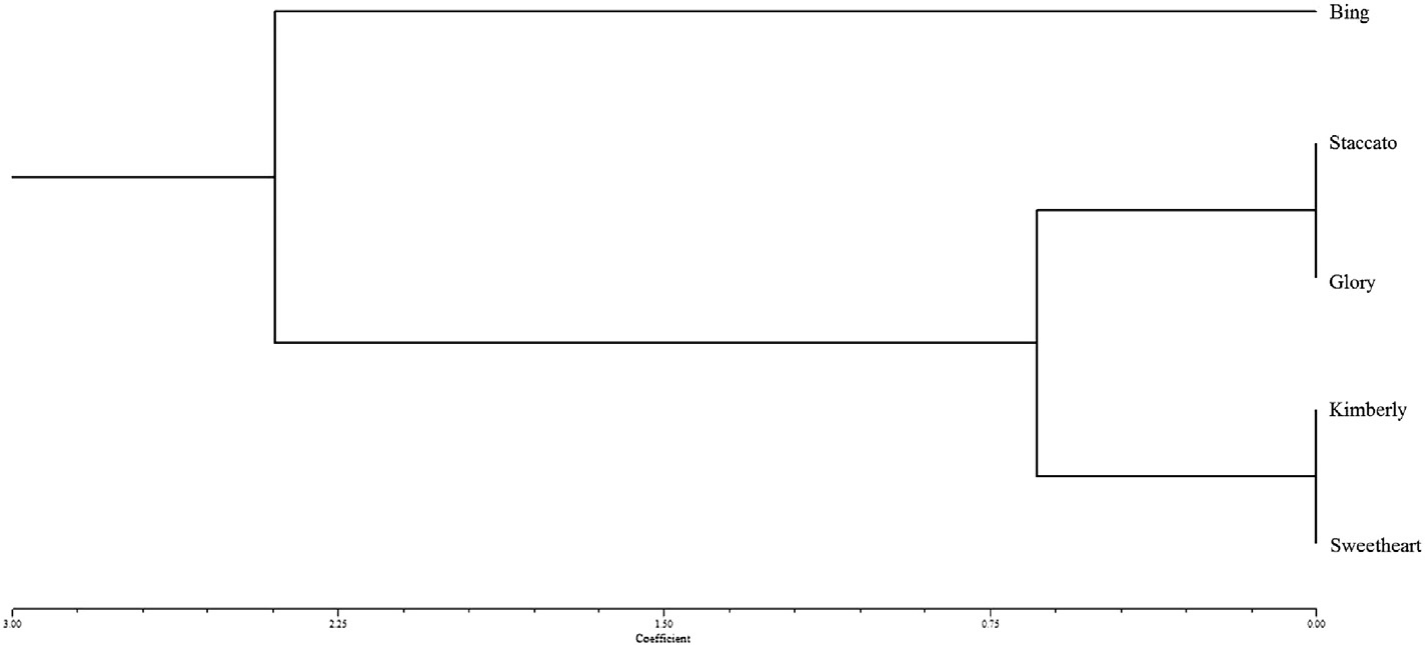
Tree dendrogram generated from SNParray data. 1385 polymorphic loci in an array of with 5696 loci were not able to distinguish between ‘Staccato’ vs. ‘Glory’ and ‘Kimberly’ vs. ‘Sweetheart’ most likely due to limited genome coverage and use of non-referenced samples resulting in ascertainment bias.

**Fig. 5:**
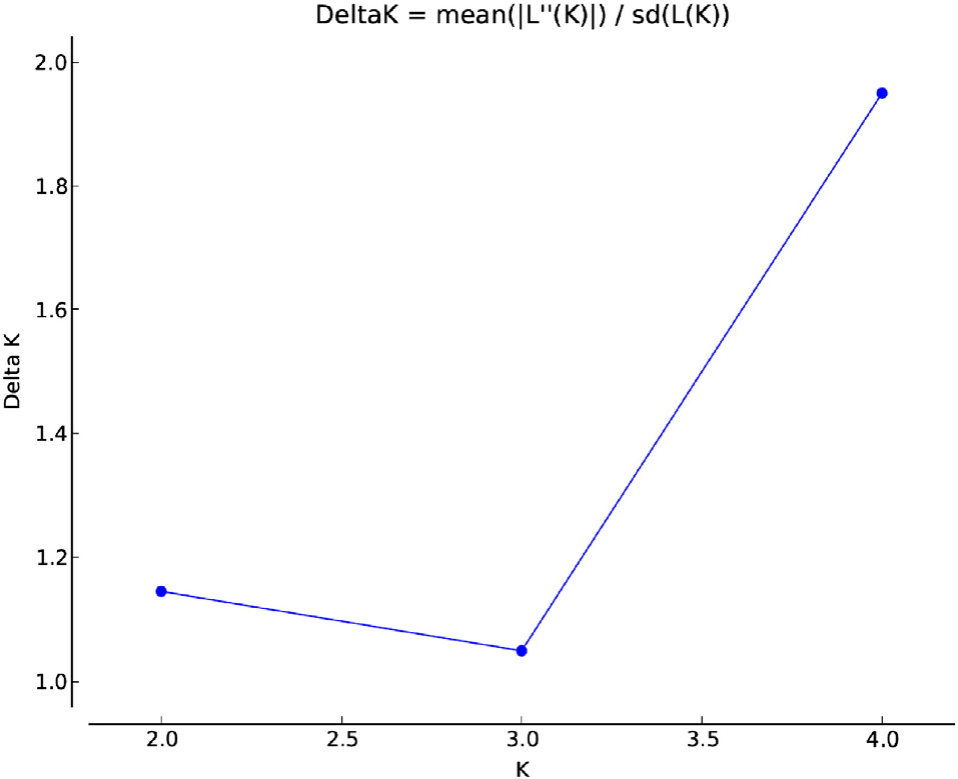
Evanno method based calculations for population number ΔK. ΔK values were highest for K = 2 and K = 4 indicating greatest likelihood of two larger groups comprised of 4 distinct subgroups.

**Fig. 6:**
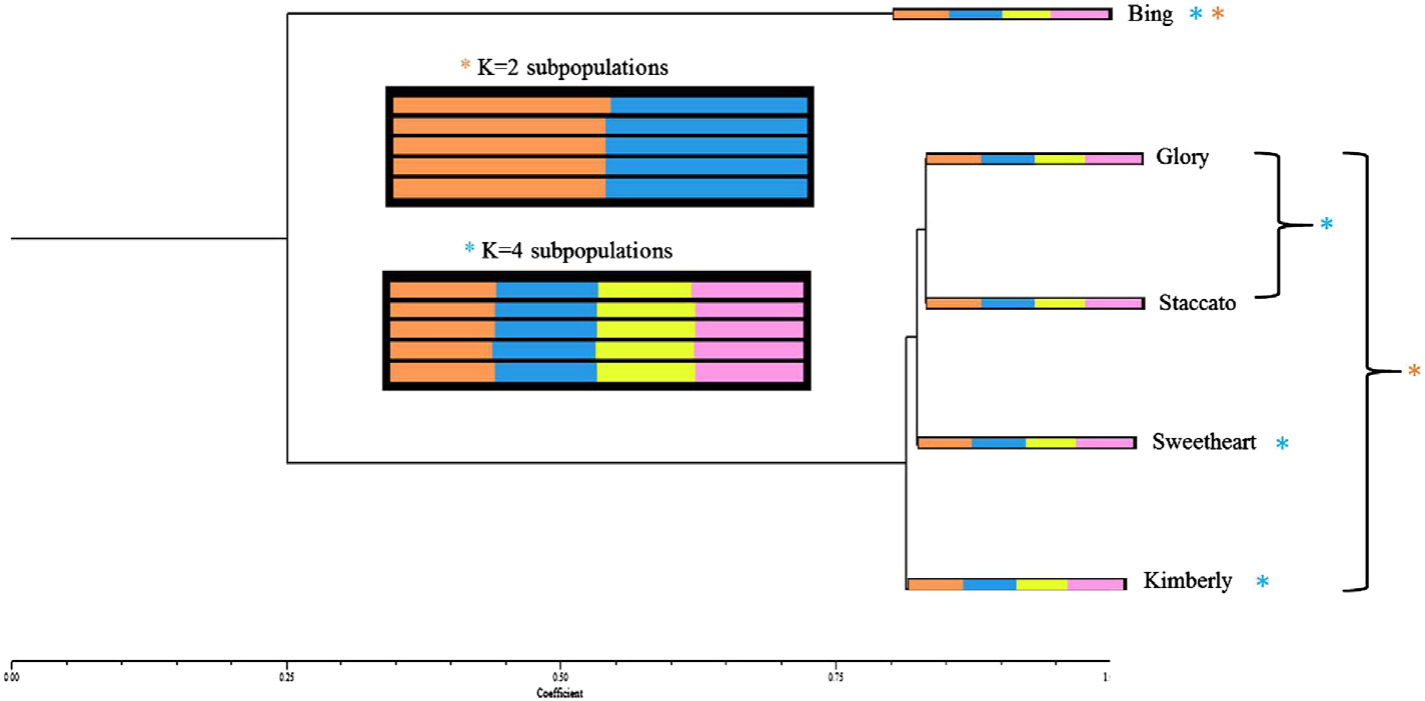
Dendrogram depicting genetic relatedness of Bing, Glory, Staccato, Sweetheart, and Kimberly based on 18,058 unique SNPs (dendrogram generated from NTSys). Colored bars represent proportion of an individual belonging to a distinct group or subgroup, based on shared and unique SNPs (generated using STRUCTURE, CLUMPP, DISTRUCT, and NTSys). Orange asterisks denote the two larger groups, and blue asterisks denote the four distinct subgroups.

STRUCTURE and NTSys were used to analyze and produce graphical representations of population structure respectively (Fig. 4, Fig. 6). In the case of both SNParray and WGS, ‘Bing’ forms an outgroup relative to the other four genotypes, which display much higher genetic similarity. This is consistent with the results of shared and unique SNP counts (Table 2) where ‘Bing’ displayed the greatest number of unique SNPs, whereas ‘Glory’, ‘Staccato’, ‘Sweetheart’, and ‘Kimberly’ possessed far fewer (0 in the case of the SNParray). While both approaches produced similar results, the greater efficiency of polymorphism detection of the WGS approach is evident. Using this method, combined with the STRUCTURE and Structure Harvester analyses, we identified 4 distinct subgroups (‘Bing’, ‘Glory’/’Staccato’, ‘Sweetheart’, ‘Kimberly’) within two larger groups; group 1 represented by ‘Bing’ and group 2 represented by ‘Glory’, ‘Staccato’, ‘Sweetheart’ and ‘Kimberly’, as shown in the ΔK graph (Fig. 5). The data from SNParray produced a similar cluster dendrogram as did the WGS approach; however STRUCTURE did not resolve differences between ‘Glory’/‘Staccato’ and ‘Sweetheart’/‘Kimberly’ in case of SNParray.

## 4 Conclusion

Multiple methods of polymorphism detection were evaluated across five closely related genotypes of sweet cherry. Each of the described approaches resulted in detection of polymorphisms, although certain ones provided higher resolution of detection between closely related genotypes.

The TRAP method allowed for identification of polymorphic regions between ‘Glory’ and ‘Staccato’. This represents the first gel-based evidence of genetic differences between these two genotypes, which were previously only distinguished by delayed fruit maturity phenotype. The observed 4.4% rate of polymorphism detection, however, is not necessarily representative of the detection rate for the TRAP approach in general. The efficiency of polymorphism identification for this method is largely dependent upon both the genetic similarity of cultivars tested as well as the specificity of the fixed primer target. While it has been demonstrated that polymorphic regions can be detected even among highly genetically similar cultivars, this success was largely dependent upon the design of primers targeting the flowering-related VRN2 gene. We recommend a primer screen of various putative gene targets in order to identify the most promising fixed primer candidates for this analysis.

The TRAPseq approach allowed for identification of 942 polymorphisms between ‘Glory’ and ‘Staccato’ using Stacks [35], [57]. As with the gel-based TRAP approach, fixed primer design is an important factor for consideration; however, TRAPseq is expected to have a broader genomic range of SNP detection when the fixed primers are designed to target diverse and rapidly evolving gene families, such as the MADS-box and PPR1 and PPR2 gene families. These genes are known to be widely distributed across the genome and represent a large family across the plant kingdom [33], [34] which are likely to contain polymorphisms when comparing closely related species. Many MADS-box genes have arisen via duplication events and have since acquired new functions [59]. Among the acquired functions is regulation of endodormancy release [60] which makes the MADS-box genes particularly useful in comparing the selected cultivars as the genotypes exhibit a late fruit maturation phenotype. Because this is a sequence based method, single nucleotide polymorphisms, which may not be visible using the gel-based approach, can be easily detected. The application of the Stacks program following sequencing of the TRAPseq PCR product allowed us to consider only those fragments that contained putative SNPs. Even though TRAPseq analysis only allows for a representation of specific primer targets throughout the genome, our evaluation demonstrates that it is able to generate quality data to identify polymorphisms between highly similar genotypes, with an observed detection rate of 3.8%.

The cherry SNParray represented 5696 SNPs derived from sweet and sour cherry accessions [31]. This method facilitated detection of 1385 SNPs when ‘Bing’, ‘Glory’, ‘Staccato’, ‘Sweetheart’ and ‘Kimberly’ genotypes were considered, an overall SNP detection rate of 24.32%. This appears far more efficient than either the gel-based TRAP approach or the TRAPseq approach. However, due to the inherent limitations of only detecting fixed, representative polymorphisms and ascertainment bias introduced due to analysis of non-referenced samples [56], the SNParray failed to identify SNPs present in the closely related genotypes. This is evident by the lack of SNP detection when ‘Glory’/‘Staccato’ and ‘Kimberly’/ ‘Sweetheart’ were compared. In such cases, a gel-based, reduced representation, and/or WGS based approach were more informative.

The WGS approach, not surprisingly, provided the highest resolution of polymorphism detection among the five genotypes analyzed. This method is advantageous in that it provides genome wide coverage and can be easily implemented in species with little or no genomic information. WGS can be limited by the depth of coverage and assembly methodology. This is especially true around polymorphic repeat regions of the genome. However, when combined with the Stacks short-read approach, the effectiveness of polymorphism detection of the WGS approach greatly increases. Processing of short reads in Stacks allowed the consideration of only regions with putative polymorphisms, which could then be used in population structure analysis of the five genotypes.

‘Bing’ is the most genetically distinct from the other genotypes analyzed, as supported by the results of NTSys, STRUCTURE, and the R clustering algorithms (Fig. 4, Fig. 6). This was expected, as more unique SNPs (almost twice as many) were identified for Bing than for any of the other cultivars analyzed (Table 2). The STRUCTURE and NTSys analyses of WGS data suggest that ‘Glory’ and ‘Staccato’ segregate together into their own subgroup, despite displaying high degree of genetic similarity to both ‘Sweetheart’ and ‘Kimberly’ (Fig. 6).

The only previously described difference between ‘Glory’ and ‘Staccato’ is based on phenotypic observation of delayed fruit maturity. Using three different methods, TRAP, TRAPseq, and WGS, it has been demonstrated that these two genotypes are subtly distinct from one another and ‘Glory’ is most likely a spontaneous mutation or ‘sport’ derived from Staccato. Thus, it seems that ‘Glory’ and ‘Staccato’, despite their high genetic similarity, are indeed distinct genotypes. Further analysis will allow us to determine whether polymorphisms between ‘Glory’ and ‘Staccato’ arose from a mutation(s) in a flowering related gene(s), as is suggested by the TRAP assay.

In summary, the sequencing based approaches evaluated in this study have generated a robust dataset of predicted polymorphisms in sweet cherry. We expect that the described methods, used in conjunction with one another, will be highly useful in genetics and genomics –based research in other closely related species of agronomic importance.

The following are the Supplementary data related to this article.

Supplementary File 1 TRAPseq loci from Stacks output. Supplementary File 1.

Supplementary File 2 Top Blast Hits for TRAPseq loci (Blast2GO). Supplementary File 2.

Supplementary File 3 Verification of SNParray derived polymorphisms. Supplementary File 3.

Supplementary File 4 SNParray STRUCTURE input file. Supplementary File 4.

Supplementary File 5 Original SNParray data. Supplementary File 5.

Supplementary File 6 Preparation of WGS Stacks output for STRUCTURE and NTSys. Supplementary File 6.

Supplementary File 7 WGS STRUCTURE input file. Supplementary File 7.

Supplementary File 8 SNParray NTSys input file. Supplementary File 8.

Supplementary File 9 WGS NTSys input file. Supplementary File 9.

Supplementary File 10 Validation of NTSys output for SNParray using pairwise SNP counts. Supplementary File 10.

Supplementary File 11 Validation of NTSys output for WGS using pairwise SNP counts. Supplementary File 11.

Supplementary File 12 Top Blast Hits for WGS loci (Blast2GO). Supplementary File 12.

## Competing Interests

The authors declare no competing interests.

## Authors' Contributions

Conceived and designed the experiments: AD, BK, SH, TK, RS.

Performed the experiments: SH, BK, TK.

Analyzed the data: SH, RH, BK, TK, AD.

Contributed reagents/materials/analysis tools: AD, RS, TK.

Wrote the paper: AD, SH, BK, RH.

## Figures and Tables

**Table 1:** TRAP and TRAPseq primers. Information regarding method, genomic target, primer type, and nucleotide sequence are provided.

Name	Method	Target	Type	Sequence
BKP-383	TRAP	VRN2	Fixed	GCGCCAATTCCAAATACAGT
BKP-384	TRAP	VRN2	Fixed	TTTTGTGACCCAATTCGACA
SA12	TRAP	–	Arbitrary	AminoC6 + DY78…TAATCCAACAACA
GA5	TRAP/TRAPseq	–	Arbitrary	AminoC6 + DY68…AAACACACATGAAGA
MADS-box	TRAPseq	MADS-box gene family	Fixed	TGGCCTCTTCAAGAAGGC
PPR1	TRAPseq	Pentatricopeptide repeat 1 gene family	Fixed	ATGGTTGATCTTCTTGGC
PPR2	TRAPseq	Pentatricopeptide repeat 2 gene family	Fixed	AATGATTGGGCGAAGGC
ODD15	TRAPseq	–	Arbitrary	AminoC6 + DY…GGATGCTACTGGTT

**Table 2:** Shared and unique SNPs identified using SNParray and WGS methods. Pairwise SNP comparison (top left) and number of unique SNPs (top right) for five sweet cherry genotypes analyzed using WGS approaches. Pairwise SNP comparison (bottom left) and number of unique SNPs (bottom right) for the five sweet cherry genotypes analyzed in the SNParray. Using the latter method, no SNPs were found between ‘Glory’ and ‘Staccato’ or ‘Kimberly’ and ‘Sweetheart’.

WGS pairwise SNP comparison						WGS unique SNPs	
	Bing	Glory	Staccato	Sweetheart	Kimberly		
Bing	0	2251	2150	2142	2217	Bing	956
Glory	2251	0	1569	1665	1771	Kimberly	496
Staccato	2150	1569	0	1620	1704	Glory	450
Sweetheart	2142	1665	1620	0	1701	Staccato	436
Kimberly	2217	1771	1704	1701	0	Sweetheart	390


SNParray pairwise SNP comparison						SNParray unique SNPs	
	Bing	Glory	Staccato	Sweetheart	Kimberly		
Bing	0	600	600	559	559	Bing	174
Glory	600	0	0	66	66	Glory	0
Staccato	600	0	0	66	66	Staccato	0
Sweetheart	559	66	66	0	0	Sweetheart	0
Kimberly	559	66	66	0	0	Kimberly	0

**Table 3:** Summary of methods employed in genome-wide polymorphism detection. Total number of loci, number of identified polymorphisms, detection efficiency and percentage genome coverage for each method (total loci sampled per 250 MB estimated genome size) were calculated for each method.

	TRAP	TRAPseq	SNParray	WGS
Samples analyzed	Glory	Glory	Glory	Glory
	Staccato	Staccato	Staccato	Staccato
			Sweetheart	Sweetheart
			Bing	Bing
			Kimberly	Kimberly
Total loci sampled	45	24984	5696	1239693
Polymorphic loci identified	2	942	1385	2071
Detection efficiency	4.44%	3.77%	24.32%	0.17%
% genome coverage Total loci sampled/250 MB (estimated genome size)	0.00000018	0.009994	0.00002278	0.00495877
