# Supplementary material for: Evaluation of multiple approaches to identify genome-wide polymorphisms in closely related genotypes of sweet cherry (Prunus avium L.)
Source: Comput Struct Biotechnol J. 2017 Mar 18;15:290–8. doi: 10.1016/j.csbj.2017.03.002 (PMC5376269; doi:10.1016/j.csbj.2017.03.002)
Supplement: Supplementary File 3 — Verification of SNParray derived polymorphisms. [file mmc3.pdf]

NCBI SS#	Original Full Name in 6K SNP Array	SNP Sequence (NCBI)	Sequence (BLAST sequence, Bing)	Predicted SNP	Actual SNP	Chromosome	Physical position (bp[a])	Estimated genetic position #1 (cM)	Estimated genetic position #2 (cM)
ss490545369	RosBREED_snp_sweet_cherry_Pp1_00094685	TTCAGTAGAGCTCCCTGGGGCTTGC[A/C]AAGTTTGTCAAACTCAATCAACTTG	TTCAGTAGAGCTCCCTGGGGCTTGCCAAGTTTGTCAAACTCAATCAACTTG	C	C	1	94685	0.21	0.00
ss490545372	RosBREED_snp_sweet_cherry_Pp1_00114487	AACAACTACCATAACAGACCTTCGA[C/T]GATAGTATACAACAAACGAAACCAC	AACAACTACCATAACAGACCTTCGATGATAGTATACAACAAACGAAACCAC	T	T	1	114487	0.26	0.00
ss490545375	RosBREED_snp_sweet_cherry_Pp1_00154827	TTGCTTTGTAGACCTTGTCCATCTA[C/T]CTTGTAGTTTTCTTCTTTTCATTAA	TTGCTTTGTAGACCTTGTCCATCTATCTTGTAGTTTTCTTCTTTTCATTAA	T	T	1	154827	0.35	0.00
ss490545378	RosBREED_snp_sweet_cherry_Pp1_00196391	TTTGGAATGTTCTTGCTCCAATTGA[G/T]CTTCTCAGCTTTGGCAGTAGTTCTC	TTTGGAATGTTCTTGCTCCAATTGATCTTCTCAGCTTTGGCAGTAGTTCTC	T	T	1	196391	0.44	0.00
ss490548745	RosBREED_snp_sweet_cherry_Pp2_00911589	ACACCCATACCCAAGTTCTTCCGTC[A/G]GAAGATGATGATGGTGCTAAGAATA	ACACCCATACCCAAGTTCTTCCGTCGGAAGATGATGATGGTGCTAAGAATA	G	G	2	911589	1.94	0.00
ss490548749	RosBREED_snp_sweet_cherry_Pp2_00979200	AAGACGGATAGCCAGGGTGAAAAAA[A/C]CTTGCCAAGTAACTAATTAATAGCA	AAGACGGATAGCCAGGGTGAAAAAACCTTGCCAAGTAACTAATTAATAGCA	C	C	2	979200	2.08	0.00
ss490548753	RosBREED_snp_sweet_cherry_Pp2_01029506	GGTTTTGTAAGGATGGTATACCTTA[C/T]TGGGAAAAGCAATTCTGCACTTTGG	GGTTTTGTAAGGATGGTATACCTTATTGGGAAAAGCAATTCTGCACTTTGG	T	T	2	1029506	2.19	0.00
ss490548757	RosBREED_snp_sweet_cherry_Pp2_01123211	TGTGTTTAACAACTTTGTCCTTGCA[A/C]AGTTTAACTGGGCAACAATATACTG	TGTGTTTAACAACTTTGTCCTTGCACAGTTTAACTGGGCAACAATATACTG	C	C	2	1123211	2.39	0.00
ss490552278	RosBREED_snp_sweet_cherry_Pp4_00355217	TAACTTCTTGCATCTTGAGGAAAAC[A/G]GGTGGTGGTAACCTAATCTCGCTGC	TAACTTCTTGCATCTTGAGGAAAACGGGTGGTGGTAACCTAATCTCGCTGC	G	G	4	355217	2.47	0.92
ss490552281	RosBREED_snp_sweet_cherry_Pp4_00394639	TTATTGCGCCAGAATCTGAGCTGAG[A/C]CGAGACGAGACTTGCCTATGGTTCA	TTATTGCGCCAGAATCTGAGCTGAGCCGAGACGAGACTTGCCTATGGTTCA	C	C	4	394639	2.58	1.07
ss490552284	RosBREED_snp_sweet_cherry_Pp4_00430644	CTGCAAAAACAACCAGCTCCGTGAA[G/T]ACATAAACACGCAGCATCCAAATGC	CTGCAAAAACAACCAGCTCCGTGAATACATAAACACGCAGCATCCAAATGC	T	T	4	430644	2.69	1.19
ss490552287	RosBREED_snp_sweet_cherry_Pp4_00473163	TCATCGGATTGGATTACCTCTCGTT[C/T]GAGTCTGAGGTGAAGGTTTATAGCC	TCATCGGATTGGATTACCTCTCGTTTGAGTCTGAGGTGAAGGTTTATAGCC	T	T	4	473163	2.81	1.35
ss490555002	RosBREED_snp_sweet_cherry_Pp6_00940675	TTCTTTACTCAGTTCTTGGTCACTG[A/C]AAAGTTCATCCGACTCTTGGTGCAC	TTCTTTACTCAGTTCTTGGTCACTGCAAAGTTCATCCGACTCTTGGTGCAC	C	C	6	940675	9.34	0.00
ss490555005	RosBREED_snp_sweet_cherry_Pp6_00981908	GATAAGTGCTGGTGAGGTTTTACAT[A/G]TCATAATATACCTGGTCTGTTTCGT	GATAAGTGCTGGTGAGGTTTTACATGTCATAATATACCTGGTCTGTTTCGT	G	G	6	981908	9.40	0.00
ss490555008	RosBREED_snp_sweet_cherry_Pp6_01023194	GGTCTATTGATTCTGGAAATGCTGC[A/G]AATGGTCAGATCCAATCTGAGCGCT	GGTCTATTGATTCTGGAAATGCTGCGAATGGTCAGATCCAATCTGAGCGCT	G	G	6	1023194	9.46	0.00
ss490555011	RosBREED_snp_sweet_cherry_Pp6_01062002	ATAGGCAACATTAAAAATATTTAAT[A/G]GGAGGCTGTTAATTCTTGCAATGCT	ATAGGCAACATTAAAAATATTTAATGGGAGGCTGTTAATTCTTGCAATGCT	G	G	6	1062002	9.51	0.00
ss490558164	RosBREED_snp_sweet_cherry_Pp8_13263864	TCTGGAAGAACTTGAAGGAATTCAC[A/G]GGTTTCAAATCCAAGAGCGGGTTGA	TCTGGAAGAACTTGAAGGAATTCACGGGTTTCAAATCCAAGAGCGGGTTGA	G	G	8	13263864	24.29	35.79
ss490558167	RosBREED_snp_sweet_cherry_Pp8_13297952	TACTAGTTTCTTCTTTTCTTTGGTC[A/G]GCATCTTCCTTGCATTCCTTATAGT	TACTAGTTTCTTCTTTTCTTTGGTCGGCATCTTCCTTGCATTCCTTATAGT	G	G	8	13297952	24.33	35.95
ss490558170	RosBREED_snp_sweet_cherry_Pp8_13376142	ATCTTTGGTGCTTTCACCATTTGAC[A/G]TGGAGAGGCTCTCTTTCTCCTCCTT	ATCTTTGGTGCTTTCACCATTTGACGTGGAGAGGCTCTCTTTCTCCTCCTT	G	G	8	13376142	24.43	36.31
ss490558173	RosBREED_snp_sweet_cherry_Pp8_13397168	TTGCAATCACTCTAATGCTTCTACT[A/C]TTTCTTGCAGATGGCCTGATCTGGA	TTGCAATCACTCTAATGCTTCTACTCTTTCTTGCAGATGGCCTGATCTGGA	C	C	8	13397168	24.46	36.41
