# Supplementary material for: Evaluation of multiple approaches to identify genome-wide polymorphisms in closely related genotypes of sweet cherry (Prunus avium L.)
Source: Comput Struct Biotechnol J. 2017 Mar 18;15:290–8. doi: 10.1016/j.csbj.2017.03.002 (PMC5376269; doi:10.1016/j.csbj.2017.03.002)
Supplement: Supplementary File 6 — Preparation of WGS Stacks output for STRUCTURE and NTSys. [file mmc6.pdf]

	8_45	14_34	18_120	20_10	21_66	23_5	24_46	34_37	35_109	40_4	43_86	47_111	48_35	51_9	54_1	55_93
Bing_NoIndex_L006_R1_001.1	1	1	4	0	4	2	0	4	0	4	2	0	0	2	3	3
Bing_NoIndex_L006_R1_001.1	3	3	4	0	4	4	0	4	0	4	4	0	0	4	4	4
Bing_NoIndex_L006_R2_001.2	0	1	4	0	4	0	1	0	1	0	0	0	1	0	3	4
Bing_NoIndex_L006_R2_001.2	0	3	4	0	4	0	3	0	4	0	0	0	4	0	4	4
Bing_NoIndex_L007_R2_002.2	0	0	4	1	2	0	1	4	0	0	0	0	4	0	0	0
Bing_NoIndex_L007_R2_002.2	0	0	4	1	4	0	1	4	0	0	0	0	4	0	0	0
Bing_NoIndex_L007_R1_002.1	0	1	0	0	0	0	1	4	1	0	4	2	0	0	3	0
Bing_NoIndex_L007_R1_002.1	0	3	0	0	0	0	3	4	4	0	4	4	0	0	4	0
Bing	1	1	4	1	4	2	1	4	1	4	2	2	1	2	3	3
Bing	3	3	4	1	4	4	3	4	4	4	4	4	4	4	4	4
Glory_NoIndex_L006_R1_001.	1	0	4	0	2	4	1	0	1	0	0	0	0	0	4	0
Glory_NoIndex_L006_R1_001.	3	0	4	0	4	4	3	0	4	0	0	0	0	0	4	0
Glory_NoIndex_L006_R2_001.	0	0	4	0	4	4	1	4	1	0	0	0	1	0	3	0
Glory_NoIndex_L006_R2_001.	0	0	4	0	4	4	1	4	4	0	0	0	4	0	4	0
Glory_NoIndex_L007_R1_002.	0	3	0	0	4	4	0	2	4	0	0	0	1	0	0	0
Glory_NoIndex_L007_R1_002.	0	3	0	0	4	4	0	4	4	0	0	0	4	0	0	0
Glory_NoIndex_L007_R2_002.	0	1	0	0	2	0	0	2	0	0	0	2	1	0	3	0
Glory_NoIndex_L007_R2_002.	0	3	0	0	4	0	0	4	0	0	0	2	4	0	4	0
Glory	1	3	4	0	2	4	1	2	1	0	0	2	1	0	3	0
Glory	3	3	4	0	4	4	3	4	4	0	0	2	4	0	4	0
Kimberly_NoIndex_L006_R1_0	0	1	0	1	0	0	1	4	4	0	0	0	1	0	0	0
Kimberly_NoIndex_L006_R1_0	0	3	0	2	0	0	3	4	4	0	0	0	4	0	0	0
Kimberly_NoIndex_L006_R2_0	0	1	0	1	2	4	1	4	0	0	0	0	0	0	0	4
Kimberly_NoIndex_L006_R2_0	0	3	0	1	4	4	1	4	0	0	0	0	0	0	0	4
Kimberly_NoIndex_L007_R1_0	1	0	0	0	0	0	1	4	4	0	2	0	0	0	0	0
Kimberly_NoIndex_L007_R1_0	3	0	0	0	0	0	1	4	4	0	4	0	0	0	0	0
Kimberly_NoIndex_L007_R2_0	1	0	4	0	4	4	1	4	1	0	0	0	4	0	0	0
Kimberly_NoIndex_L007_R2_0	3	0	4	0	4	4	1	4	4	0	0	0	4	0	0	0
Kimberly	1	1	4	1	2	4	1	4	4	0	2	0	1	0	0	4
Kimberly	3	3	4	2	4	4	1	4	4	0	4	0	4	0	0	4
