# Supplementary figures and images for: Evaluation of multiple approaches to identify genome-wide polymorphisms in closely related genotypes of sweet cherry (Prunus avium L.)
Source: Comput Struct Biotechnol J. 2017 Mar 18;15:290–8. doi: 10.1016/j.csbj.2017.03.002 (PMC5376269; doi:10.1016/j.csbj.2017.03.002)

Cluster Dendrogram

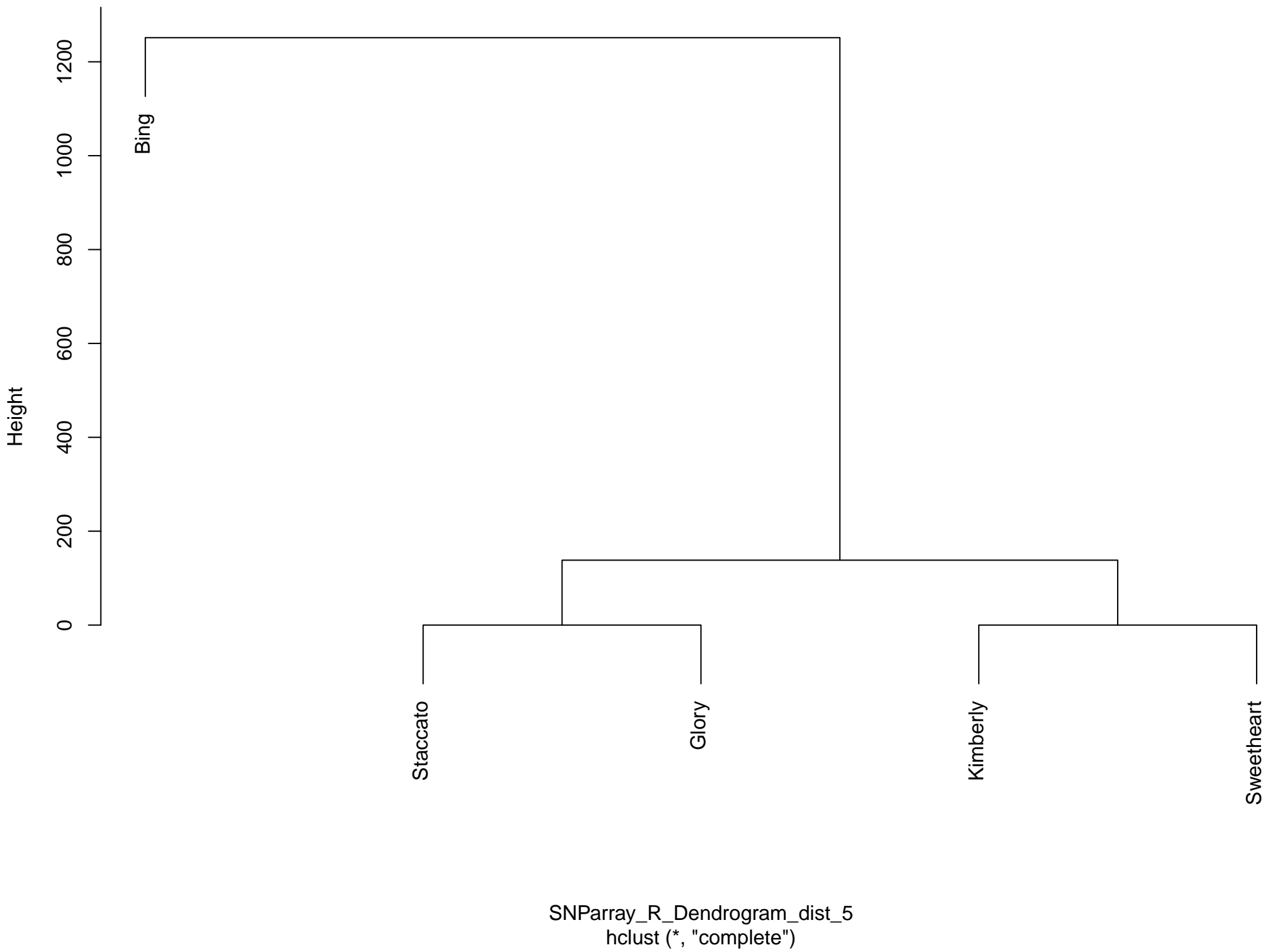

Supplement: Supplementary File 10 — Validation of NTSys output for SNParray using pairwise SNP counts. [file mmc10.pdf]

Cluster Dendrogram

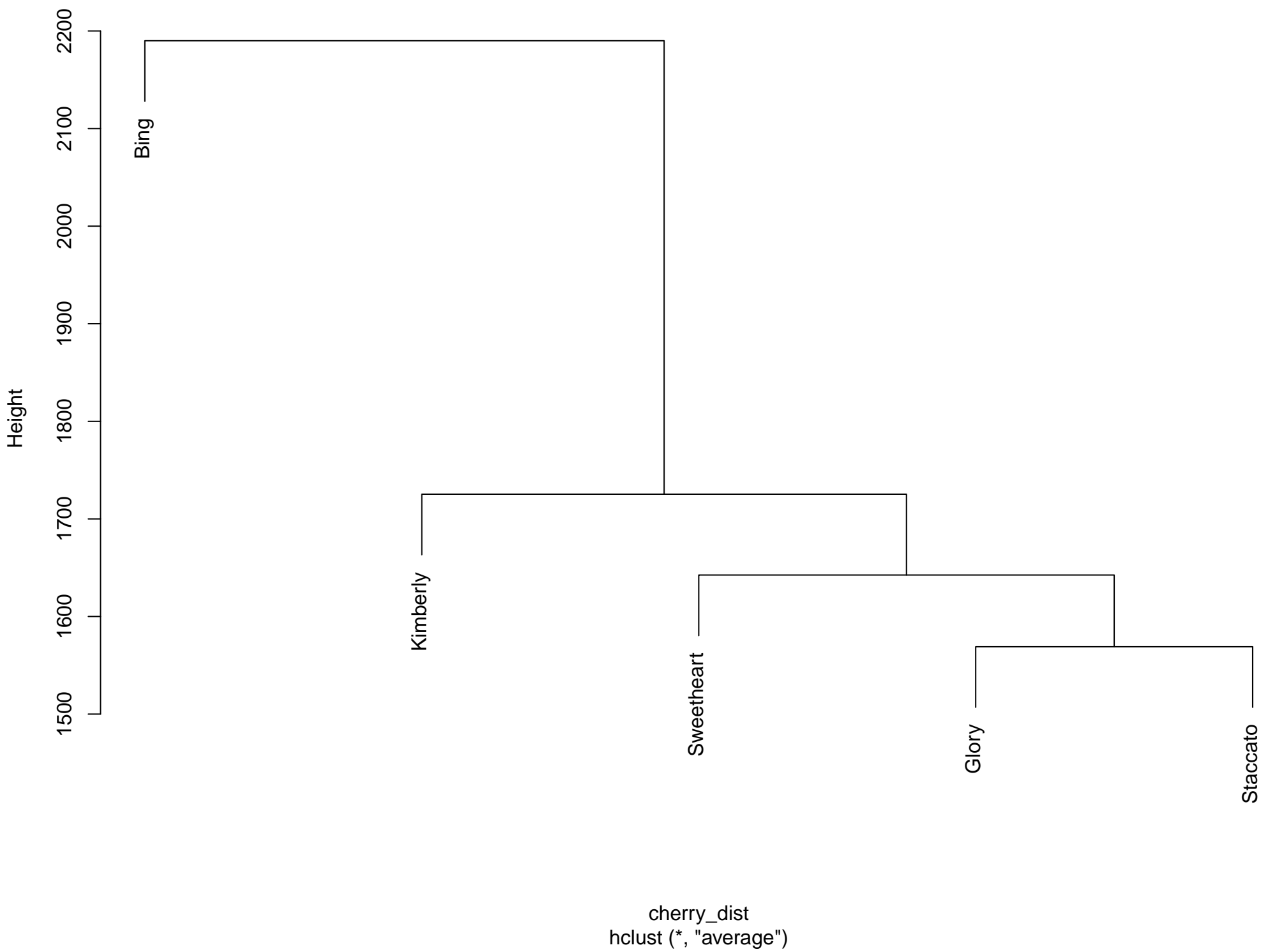

Supplement: Supplementary File 11 — Validation of NTSys output for WGS using pairwise SNP counts. [file mmc11.pdf]
